# Dietary Intake and Content of Cu, Mn, Fe, and Zn in Selected Cereal Products Marketed in Poland

**DOI:** 10.1007/s12011-018-1384-0

**Published:** 2018-05-18

**Authors:** Anna Winiarska-Mieczan, Edyta Kowalczuk-Vasilev, Katarzyna Kwiatkowska, Małgorzata Kwiecień, Ewa Baranowska-Wójcik, Bożena Kiczorowska, Renata Klebaniuk, Wioletta Samolińska

**Affiliations:** 1 Department of Bromatology and Food Physiology, University of Life Sciences in Lublin, Akademicka 13, 20-950 Lublin, Poland; 2 Department of Biotechnology, Microbiology and Human Nutrition, University of Life Sciences in Lublin, Lublin, Poland

**Keywords:** Cereal products, Cu, Mn, Fe, Zn, Intake, Reference daily dose

## Abstract

This paper aims to verify whether cereal products are a good source of Cu, Mn, Fe, and Zn in the diets of Poles. The study material comprised of 445 cereal products including baked goods, breakfast cereals and groats, pasta, and rice. Products that required culinary treatment (pasta, groats, rice) were boiled in drinking quality water as recommended by the producer. The content of Cu, Mn, Fe, and Zn was determined by using the FAAS method. The average content of microelements in the analyzed products can be represented as Fe (17.9 mg kg^−1^ ± 10.3) > Zn (12.4 mg kg^−1^ ± 5.2) > Mn (9.6 mg kg^−1^ ± 6.7) > Cu (3.9 mg kg^−1^ ± 2.9). Considering the daily requirement of the analyzed minerals among adult Poles, it was determined that cereal products supply 58% RDA Cu, 61% AI Mn (men)–78% AI Mn (women), 19% RDA Fe (women)–34% RDA Fe (men), and 16% RDA Zn (men)–22% RDA Fe (women). Baked goods account for as much as about 90% of all Cu, Mn, Fe, and Zn consumed as cereal products in a daily diet. Based on the results, it can be concluded that cereal products are the main source of Cu, Mn, Fe, and Zn in the diets of Poles. In connection with low assimilability of minerals, they should not be considered the fundamental source of those microelements in the diets of Poles.

## Introduction

According to the current state of knowledge, some microelements are deemed necessary to ensure the correct development and functioning of the human body. These include copper (Cu), manganese (Mn), iron (Fe), and zinc (Zn). These elements perform different functions, e.g., they form part of compounds of fundamental significance to metabolic processes, such as enzymes and their activators and hormones. They also participate in regulating and immune mechanisms [1, 2]. Insufficient levels of Fe and Cu or/and imbalance between Fe and Cu increase the risk of developing neurological diseases such as Alzheimer’s [3]. Insufficient level of Zn in the body can lead to skeletal and reproductive system disorders, skin inflammations, atherosclerotic processes, and infections [4]. In addition, Zn deficiency concurrent with an excess of Cu is observed in autistic children [5]. Although deficiencies of Mn in the human diet throughout the world are very rare, in turn, excessive amounts of this element can pose a problem. These are, in particular, connected with the intensification of manganese ore mining and a considerable increase in the levels of Mn in air, water, and food [6]. At present, the toxicity of Mn is connected with free radical damage of cells of the extrapyramidal system, and in particular the dopamine system, which leads to developing Parkinsonism [7]. However, it must be remembered that manganese supplied with a diet is absorbed to a small extent, not exceeding 5% [8]. Therefore, excessive absorption can be observed only in people with liver disorder or damage [9].

Analyses of eating habits carried out in Poland revealed that nutritional risk factors posing hazard to the health of the population often occurred. The most frequently mentioned were insufficient level of Cu in the diets of children, young adults, and the elderly [10–12]; insufficient level of Fe, in particular, in the diets of vegans or vegetarians, people suffering from malabsorption, women with heavy periods, expectant and lactating women, and children [13–15]; and insufficient level of Zn in the diets of the elderly and young adults [10, 12]. Furthermore, analysis of the concentration of microelements in the hair of the elderly (aged 70–80) revealed Fe and Zn deficiency in nearly 40% and Cu deficiency in more than 50% of the analyzed subjects in comparison to reference values for these elements, which is connected with insufficient supply of these microelements [16]. In addition, analyses of breast milk showed low levels of Cu and Zn that did not cover the infant’s requirement of these elements, which was also connected with a deficiency of such elements in a woman’s diet [17]. At the same time, in some cases, excessive levels of these minerals were found in the diets of Poles. However, this referred to overweight people [18]. In turn, excess levels of Mn are often identified in the diets of Poles. For adults, the requirement can be exceeded by more than two times [19, 20].

Copper, Fe, and Zn deficiency and possible excessive amounts of Mn in a diet are not only applicable to Poland. Available literature indicates that an identical problem also exists in other countries all over the world [6, 21]. According to WHO [22], about 2 billion people out of the world population (30%) suffer from anemia, including half due to Fe deficiency. Insufficient intake of Fe is one of the three most frequently recorded types of mineral insufficiency in the world [23]. It is estimated that as many as 40% of the world’s population suffering from diseases connected with Zn deficiency suffer from a nutritional deficiency of Zn [24]. Therefore, the intake of elements that are most often insufficiently supplied should be monitored on a continuous basis, which is recommended by organizations throughout the world promoting healthy nutrition [25].

Food is the basic source of minerals for humans. Foodstuffs are characterized by different content of minerals, which is connected with the type of raw material used in the production of food, and the conditions of obtaining and processing such raw materials. Cereal products are the basis in nutrition all over the world, whole grain products being particularly valuable [26]. In Poland, in 2015, the monthly intake of cereal products amounted to 5.88 kg per person, while the monthly consumption of meat was 5.32 kg, fish and seafood—0.32 kg, milk and dairy—4.82 kg, fruits—3.66 kg, and vegetables—8.59 kg [27]. Despite cereals being considered a moderately rich source of microelements [28], due to the fundamental share of cereal products in the human diet, they can be an important source of such elements. This paper aims to verify whether cereal products are a good source of Cu, Mn, Fe, and Zn in the diets of Poles. The presented work is a part of the project aiming to estimate the intake of minerals (necessary and toxic) in the Polish population. Available literature does not contain information relating to this subject.

## Material and Methods

### Study Material

The study material comprised 445 cereal products purchased from local groceries (9 supermarkets and 12 small groceries) ahead of their best-before dates (Table 1). Products that required culinary treatment (pasta, groats, rice) were boiled in drinking quality water as recommended by the producer. Prior to analyses all products were dried at 65 °C over 24 h and then also ground in an electric grinder. From each of the ground products, a sample weighing approx. 30 g was taken. The samples were put one by one in separate, numbered, tightly closed plastic containers that were stored for further chemical analyses (max. 10 days) in a dark room at room temperature.

**Table 1:** Characteristic of the analyzed products

Products	*n*	Characteristic
Breads	84	
Whole meal rye	27	With sunflower, pumpkin, flax, and/or rye seeds
Wheat	16	Without additions
Whole meal wheat	10	With sunflower, pumpkin, and/or flax seeds
Mixed wheat-rye	31	Without additions or with sunflower, pumpkin, and/or flaxseed
Rolls	36	
Rye	8	Without additions
Rye with additions	8	With sunflower and/or pumpkin seeds
Wheat	10	Without additions
Wheat with additions	10	With sunflower and/or pumpkin seeds, with dried tomatoes, olives, and/or herbs
Breakfast cereals	232	
Rice	15	Without additions
Corn	42	Without additions
Wheat	21	Without additions
Oat	13	Without additions
Whole grain and bran	41	Wheat, rye, oat
Mixed cereals	20	Wheat, rye, oat
Sweet cereals	23	With sugar, chocolate, or honey during production
With dairy	21	With dairy (milk, yoghurt) during production
Fitness	10	Whole grain (wheat, rye, oat), without additions or with yoghurt, dried fruits
Muesli i crunchy	26	Wheat, rye, oat, corn, with yoghurt, dried fruits
Pasta	39	
Wheat without additions	18	White or whole grain, durum
Wheat with additions	7	With spinach or tomatoes
Gluten free	8	Rice and corn
Rice	6	Without additions
Groats and rice	38	
Oat	15	Without additions
Buckwheat	14	Without additions
Couscous	6	Without additions
Chia	3	Without additions
Rice	26	
Rice	20	Polished, without additions
Rice	6	Brown, without additions

### Chemical Analyses

Prior to analyses, the ground material was mixed manually. Next, approx. 3 g was taken from each sample—each in three replications. All the samples were subjected to combined mineralization in a muffle furnace at 450 °C for 12 h with H_2_O_2_ used as an oxidant. The sample burning procedure was repeated four times until they turned into white ash. Next, the ash was dissolved in 10 cm^3^ 1 M HNO_3_, as described in another paper [29]. The content of Cu, Mn, Fe, and Zn was determined using the FAAS (flame atomic absorption spectrometry) method in a Varian SpectrAA 280 FS apparatus, using an SPS3 Autosampler (Table 2). Each analysis was carried out in three replications. The deviation in measurement did not exceed 4.8%. The rate of recovery of the analyzed minerals ranged from 91 to 106%. The results were verified by means of a blind sample (1 M HNO_3_) and certified reference materials LGC-7173 Poultry Feed, BCR-063R Skimmed Milk Powder, and SRM-1546 Meat Homogenate.

**Table 2:** Measurement parameters for the determination of Cu, Mn, Fe and Zn

	Cu	Mn	Fe	Zn
Wave length (nm)	324.8	279.5	248.3	213.9
Lamp current (mA)	3.5	5.0	5.0	3.5
Spectral band pass (nm)	0.5	0.2	0.2	1.0
LOD (mg kg^−1^)	1.3	1.1	4.3	10
LOQ (mg kg^−1^)	2.6	2.2	8.6	20
Pure gas	C_2_H_2_/Air	C_2_H_2_/Air	C_2_H_2_/Air	C_2_H_2_/Air
The deviation of duplicate measurement (%)	2.3	1.0	2.8	3.8
Reproducibility (%)	2.3	9.2	8.6	5.1
Quality control				
Blank sample	1 M HNO_3_	1 M HNO_3_	1 M HNO_3_	1 M HNO_3_
Certified reference material (1)	BCR-063R	LGC-7173	LGC-7173	LGC-7173
Certified (mg kg^−1^)	0.602	131.0	145.0	91.0
Observed (mg kg^−1^)	0.596	127.1	139.2	96.46
Recovery rate (%)	99	97	96	106
Certified reference material (2)	SRM-1546	SRM-1546	SRM-1546	SRM-1546
Certified (mg kg^−1^)	0.605	0.286	10.17	17.88
Observed (mg kg^−1^)	0.575	0.283	9.255	17.52
Recovery rate, %	95	99	91	98

### Reagents and Reference Materials

All solutions were prepared using deionized water (Hydrolab Poland, Gdańsk deionizer system) and ultra-pure chemical reagents. Hydrogen peroxide H_2_O_2_ (30% pure, catalogue number: 885193427) and nitric acid HNO_3_ (65% ultra purum, catalogue number: 529602839) were purchased from POCH S.A. (Poland). One-element standard solutions Ultra Scientific (LGC Standards Sp. z o.o., Poland) used for determining the reference curve contained 1000 mg L^−1^ Cu, Mn, Fe, and Zn each (99.99% purity). The certified reference material LGC-7173 Poultry Feed (LGC, Germany) contained 131 mg Mn, 145 mg Fe, and 91 mg Zn per 1 kg. The certified reference material BCR-063R Skimmed Milk Powder (Joint Research Centre, Belgium) contained 0.602 mg Cu per 1 kg. The certified reference material SRM-1546 Meat Homogenate (National Institute of Standards and Technology, USA) contained 0.286 mg Mn, 10.17 mg Fe, 17.88 mg Zn, and 0.605 mg Cu per 1 kg.

### Calculations

The average content of minerals in the analyzed products was calculated taking into account three replications for each determination in every sample. Because the levels of Cd and Pb were determined in dried material, the results were converted into the content of these minerals in natural mass by means of the drying ratio calculated from the formula: weight of dried sample/weight of fresh sample. Considering the average monthly consumption of respective groups of cereal products in Poland in 2016: baked goods—3.52 kg, cereals and groats—0.13 kg, pasta—0.38 kg, rice—0.16 kg (4.19 kg in total) [27] and the average content of Cu, Mn, Fe, and Zn in products, the average weekly intake of minerals with such products was calculated along with the share of such products in the supply of the analyzed minerals. The intake of microelements with cereal products was compared against Polish RDA—Recommended Dietary Allowances (Cu, Fe, and Zn) or AI—Adequate Intake (Mn) for adults (women and men) [30].

Statistical analyses of the results were performed using Statistica 6.0 software. The significance of differences between average content of microelement products from different groups was determined by one-factor variance analysis (ANOVA) using the Duncan test; *p* < 0.05 was assumed as a statistically significant level.

## Results and Discussion

### Content of Cu, Mn, Fe, and Zn in Cereal Products

The average content of the analyzed minerals in the studied products was approx. 3.9 mg Cu (range 1.108–8.906), 9.5 mg Mn (range 3.681–21.38), 17.9 mg Fe (range 4.921–29.34), and 12.4 mg Zn (range 6.347–20.24) per 1 kg of fresh weight (Table 3). The average content of microelements can be represented as Fe > Zn > Mn > Cu. The highest Fe content (*p* < 0.05) was recorded in breakfast cereals (29.3 ± 12.9 mg) and in baked goods (26 mg in rolls and buns and approx. 25 mg in bread). In addition, breakfast cereals contained significantly more Mn and Zn than other products did (Table 4). The highest content of Cu (*p* < 0.05) was noted in groats. Out of the analyzed products, rice contained the lowest amounts of all the analyzed microelements (*p* < 0.05).

**Table 3:** The content of Cu, Mn, Fe, and Zn in analyzed cereal products, mg kg^−1^ fresh weight

	*n*	Cu	Mn	Fe	Zn
Breads	84	3.462 ± 1.017	13.05 ± 6.057	24.95 ± 11.40	12.68 ± 4.183
Rolls	36	4.069 ± 0.497	7.286 ± 3.127	26.08 ± 11.40	12.22 ± 4.914
Breakfast cereals	232	4.496 ± 1.555	21.38 ± 18.12	29.34 ± 12.91	20.24 ± 11.69
Pasta*	39	1.108 ± 0.540	3.681 ± 2.897	8.002 ± 5.574	6.347 ± 3.812
Groats*	38	8.906 ± 5.144	7.728 ± 4.172	13.97 ± 8.460	15.60 ± 7.855
Rice*	26	1.153 ± 0.546	3.924 ± 0.240	4.921 ± 0.261	7.128 ± 0.225
Arithmetic mean		3.866^D^	9.508^C^	17.88^A^	12.37^B^
Maximum		8.906	21.38	29.34	20.24
Minimum		1.108	3.681	4.921	6.347
SD		2.863	6.734	10.29	5.220
Median		3.766	7.507	19.46	12.45

Average values for samples, each in three replications. *SD* standard deviation. *Cooked pasta, groats, and rice (boiled without salt). Different superscripts in the same line differ at *p* < 0.05 by Duncan’s test

**Table 4:** Statistical analyses of the results

	Cu	Mn	Fe	Zn	*p*
Breads	c D	b B	b A	c C	0.002
Rolls	b D	c C	b A	c B	0.002
Breakfast cereals	b D	a B	a A	a C	0.001
Pasta*	d D	d C	d A	e B	0.031
Groats*	a C	c D	c B	b A	0.007
Rice*	d D	d C	e B	d A	0.011
p	0.011	0.006	0.008	0.031	

Different lowercase letters in the same column differ at *p* < 0.05 by Duncan’s test. Different uppercase letters in the same line differ at *p* < 0.05 by Duncan’s test. *cooked pasta, groats, and rice (boiled without salt)

The content of microelements in cereal products is mostly determined by the type of cereal grain and/or composition of flour used for the production. Minerals are mostly contained in the aleurone layer of the grain [31]; thus, whole meal products are richer in such elements [32]. Wheat plays the most important role in the diets of Europeans, whereas more than half of the world’s population eats mostly rice, and mainly polished rice [33]. The grains of wheat and rice contain less microelements than other cereal grains [33]. To some extent, the variety of cereal grain and the richness of soil also determine the content of microelements in cereal grains, which was demonstrated, for instance by the studies into different genetic lines of wheat [34]. In turn, the high content of microelements in cereal products with an addition of, e.g., honey, nuts, seeds, or dried fruit results from the natural high content of such microelements in the additions [35].

### Baked Goods

In the presented study, the average content of microelements in bread and in rolls was established, and it was determined that baked goods contained Fe > Zn > Mn > Cu. This relationship was different in bread and in rolls. In the presented study, rolls contained 4.07 ± 1.0 mg Cu, 7.3 ± 3.1 mg Mn, 26.1 ± 11.4 mg Fe, and 12.2 ± 4.9 mg Zn per 1 kg (Table 3): Fe > Zn > Mn > Cu. In comparison to bread, the rolls contained less Mn than Zn, which was likely to have been connected with the components of the baked goods. On the other hand, in the presented study, bread contained on average ca. 3.5 ± 1.0 mg Cu, 13 ± 6.1 mg Mn, 25 ± 11.4 mg Fe, and 12.7 ± 4.2 mg Zn per 1 kg of fresh weight (Table 3). This relationship can be represented as Fe > Zn > Mn > Cu. Also, previous studies involving 10 types of Polish bread showed an identical relationship [36]. Similarly, Khouzam et al. [37] demonstrated that in Lebanese bread, the relationship between the content of microelements can be represented as Fe > Mn > Zn > Cu. Their values were 1.7–6 mg Cu, 8.2–40.9 mg Zn, 7.5–58.7 mg Mn, and 15.7–59.4 mg Fe per 1 kg, respectively. Also, for the bread from Nigeria, the relationship was Mn > Zn > Cu [38]. Al-Mussali and Al-Gahri [39], having analyzed 10 types of bread consumed in Yemen, found that 1 kg contained 19.9–78.2 mg Fe and 7.78–20.4 mg Zn, which, considering the maximum values, also supports the abovementioned relationship. At the same time, those authors noted that dark bread contained more of such microelements than white bread.

### Breakfast Cereals

The analyzed breakfast cereals contained on average 4.5 ± 1.6 mg Cu, 21.4 ± 18.1 mg Mn, 29.3 ± 12.9 mg Fe, and 20.2 ± 11.7 mg Zn per 1 kg of fresh weight of the product (Table 3), which can be represented as Fe > Mn > Zn > Cu. Studies carried out in Poland and presented by Leśniewicz et al. [40] showed an identical relationship. According to those authors, breakfast cereals contained on average 1.67–167 mg Fe, 0.34–15.8 mg Mn, 0.21–14.4 mg Zn, and 0.10–3.67 mg Cu per 1 kg. Studies carried out by the same authors [41], the results of which were published in 2012, also supported this relationship. Similarly, the analysis of the content of minerals in various kinds of granola, carried out by Eke-Ejiofor and Beleya [42], showed Fe (58.8–73.7 mg kg^−1^) > Zn (15.3–22.7 mg kg^−1^) > Cu (2.1–4.7 mg kg^−1^).

### Pasta

In the presented studies, the analysis of boiled pasta showed the following content of microelements: Fe > Zn > Mn > Cu. These values amounted to 8.0 ± 5.57 mg kg^−1^ Fe, 6.35 ± 3.81 mg kg^−1^ Zn, 3.68 ± 2.9 mg kg^−1^ Mn, and 1.11 ± 0.54 mg kg^−1^ Cu (Table 3). Similarly, Kunachowicz et al. [35] demonstrated that in boiled pasta, the relationship between the content of microelements can be represented as Fe > Zn > Mn > Cu. In turn, studies carried out in the USA by Albrecht et al. [43] showed that 1 kg of pasta boiled in unsalted water contained 9–13 mg Fe, 6–7 mg Zn, and 2–4 mg Mn. The level of Cu was lower than determinable. On the other hand, pasta boiled in salted water contained 17–21 mg Fe, 7–10 mg Zn, 3–5 mg Mn, and 2 mg Cu (relationship between the analyzed microelements: Fe > Zn > Mn > Cu). According to Cubadda et al. [44], boiled Italian durum wheat pasta contained 5.1–6.3 mg Fe, 4.4–5.2 mg Zn, and 0.98–1.13 mg Cu per 1 kg (relationship, Fe > Zn > Mn > Cu). However, Serbian studies revealed that boiled whole-wheat pasta contained approx. 6.15 mg Fe, 2.63 mg Mn, 2.23 mg Zn, and 0.34 mg Cu, which means that in that case, the content of Mn was higher than the content of Zn [45]. It is reasonable since whole-wheat flour contains more Mn than Zn, whereas semolina, which is most often used in the production of pasta, contains more Zn than Mn [32].

When pasta is boiled, certain amounts of minerals pass into the water and/or are absorbed from the water [43]. However, according to these authors, the rate of retention of Fe, Zn, Mn, and Cu is high (more than 86%), irrespective of whether the pasta was boiled in drinking or distilled water and whether the water was salted. It should be noted that for raw (dry) pasta, the relationship between the analyzed microelements was identical to that in boiled pasta: Fe > Zn > Mn > Cu [44].

### Groats

**Fig. 1:**
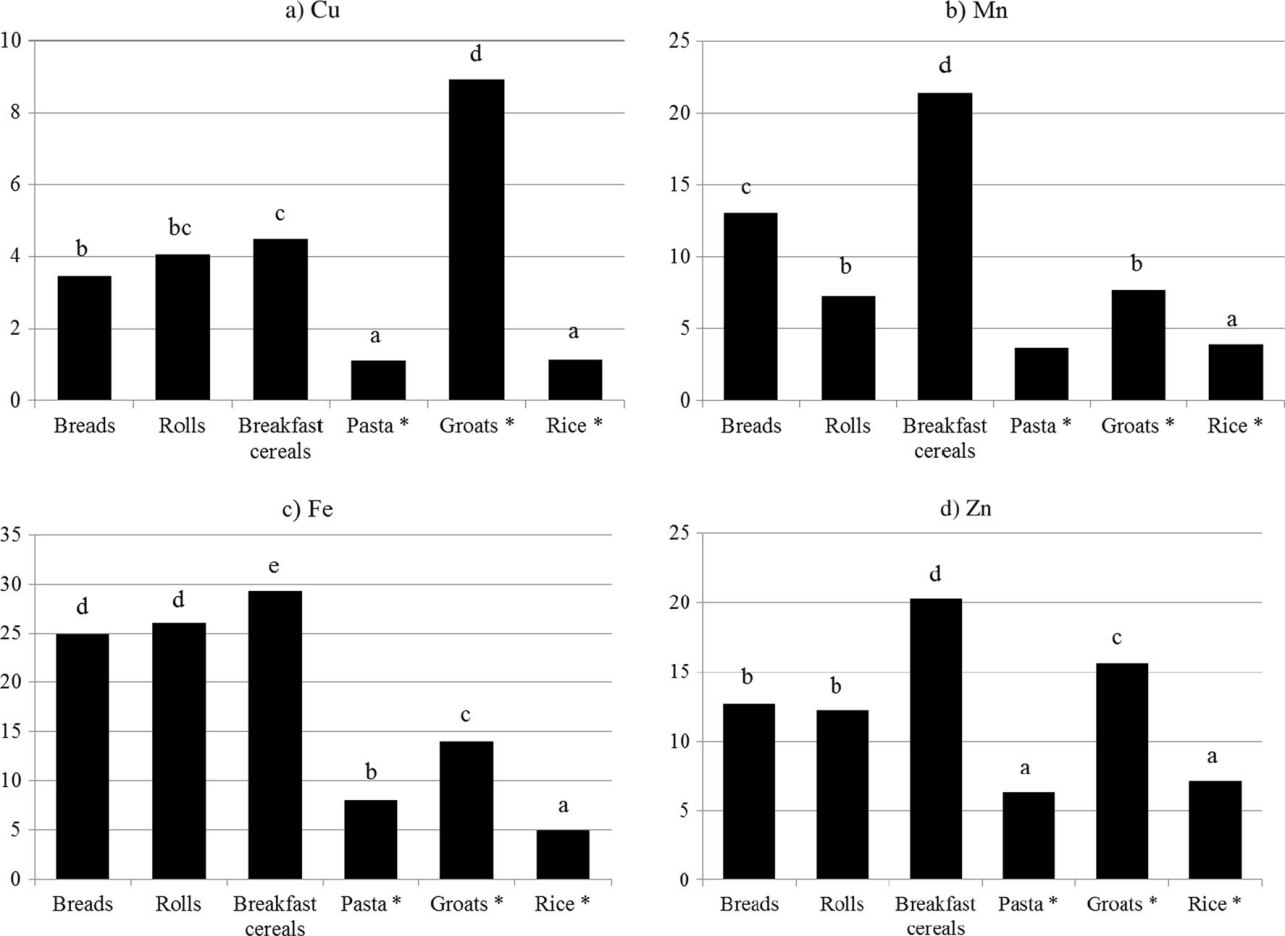
The content of **a** Cu, **b** Mn, **c** Fe, and **d** Zn in analyzed cereal products, mg kg^−1^ dry matter. *cooked pasta, groats, and rice. a, b, c, d, e are values that differ at *p* < 0.05 by Duncan’s test

In the presented studies, for boiled groats it was demonstrated that Zn > Fe > Cu > Mn (Table 3, Fig. 1). The respective values per 1 kg of fresh weight were 16.6 ± 7.9 mg Zn, 13.97 ± 8.5 mg Fe, 8.91 ± 5.14 mg Cu, and 7.73 ± 4.15 mg Mn. Available literature contains little information on the content of microelements in boiled groats. However, studies by Ikeda et al. [46] revealed that the grains of boiled buckwheat retain nearly 80% Cu and more than 95% Zn and Mn. According to Kunachowicz et al. [35] boiled buckwheat and millet groats contained Zn > Fe > Mn > Cu, whereas barley groats Fe > Zn > Mn > Cu. Groats are characterized by different mineral composition, depending on the variety of cereal grain from which they are produced. The richest source of microelements is buckwheat—Zn and Cu it contains are characterized by particularly high bioavailability [47].

### Rice

In the presented studies, boiled rice contained Zn > Fe > Mn > Cu. The relationship was noted both for fresh and dry weight (Fig. 1). The respective values amounted to 7.13 ± 0.23 mg Zn, 4.92 ± 0.26 mg Fe, 3.92 ± 0.24 mg Mn, and 1.15 ± 0.55 mg Cu per 1 kg of fresh weight (Table 3). Available literature contains little data on the content of microelements in boiled rice. However, studies by Ziarati and Azizi [48] showed that boiling induces a statistically significant reduction in the content of Zn, Mn, and Fe compared to raw grains, which is connected with the passing of these minerals into water. It was demonstrated that even soaking the grains for 1.5 h at 40 °C induces a statistically significant reduction in their Fe, Zn, and Mn content as a result of flushing. In addition, a decrease in the level of minerals was noted along with the increase in soaking temperature [49]. Omar et al. [50] reported that the relationship for boiled rice was Zn > Fe > Cu. Tegegne et al. [51] presented the content of microelements in boiled rice. The results were calculated per dry weight of the product. Those authors determined the following relationship: Fe > Zn > Cu > Mn. According to Kunachowicz et al. [35], boiled white rice contained Zn > Mn > Fe > Cu. Differences between results obtained in studies carried out by the present authors (Zn > Fe > Mn > Cu) and those presented by Tegegne et al. [51] and Kunachowicz et al. [35] can certainly be attributed to the variety of rice, since results of different studies showed that the variety had an essential effect on the mineral composition of grains. Analysis of raw Chinese rice showed a relationship between minerals which can be represented as Zn > Mn > Cu > Fe [50]. In turn, for Ethiopian rice, it was Fe > Zn > Cu > Mn [52].

### Share of Cereal Products in Supplying Cu, Mn, Fe, and Zn in the Diets of Poles

Taking the average intake of the studied cereal products by Poles (4.19 kg per month) into account, it was estimated that together with these products they consumed 0.492 mg Cu, 1.324 mg Mn, 3.215 mg Fe, and 1.657 mg Zn on a daily basis (Table 5). The share of these products in the daily supply of the analyzed minerals is approx. 23–59% Cu, 13–35% Mn, 21–23% Fe, and 15–24% Zn (Table 5). Considering the daily requirement of the analyzed minerals among adult Poles, it was determined that cereal products supplied 55% RDA Cu, 58% AI Mn (men)–74% AI Mn (women), 18% RDA Fe (women)–32% RDA Fe (men), and 15% RDA Zn (men)–21% RDA Fe (women). Based on the results, it can be concluded that cereal products are an important source of microelements in the diets of Poles. Similar conclusions were presented by Rubio et al. [54]. According to those authors, cereal products considerably cover the microelement requirement of the Spanish, supplying 0.4 mg Cu, 0.98 mg Mn, 2.74 mg Fe, and 1.72 mg Zn per day, which regarding their daily supply accounts for ca. 19% Cu and Zn, 21% Fe, and 41% Mn. As regards the diets of Italians, cereal products (together with bulb vegetables) supply 25% Cu, 42% Fe, 26% Zn, and as much as 60% Mn [55]; whereas, other data [56] suggests that cereal products alone supply 35% Cu, 30% Fe, and 18% Zn. In the French population, the share of cereal products in the daily supply of Cu is 25–30%, while for meat supplies 15–20% Cu, fruits and vegetables ca. 15%, and dairy products only ca. 10% [57]. It is assumed that about 25% Zn and 44% Fe present in the diets of the British are derived from cereal products [58]. In the diets of the Japanese cereal products account for approx. 33% of the daily requirement of Cu, approx. 28% Zn, and approx. 32% Mn, whereas the share of fruits and vegetables does not exceed 20% Cu, 5% Zn, and 17% Mn, and the total share of meat and fish is not higher than 15% Cu, 30% Zn, and 2.5% Mn [59].

**Table 5:** Share of cereal products in the supply of Cu, Mn, Fe, and Zn in the diets of adult Poles

	Cu	Mn	Fe	Zn
Daily dietary intake (mg)^a^	0.83–2.10	3.80–10.2	13.7–15.4	6.90–11.3
Daily supply with cereal products (mg) ^b^	0.491	1.324	3.215	1.657
Share of cereal products in daily supply (%)^c^	23.38–59.16	12.98–34.83	20.88–23.47	14.67–24.02
Reference daily intake (mg)^d^				
Women	0.9	1.8	18	8
Men	0.9	2.3	10	11
Share of cereal products in reference daily intake (%)				
Women	54.56	73.54	17.86	20.72
Men	54.56	57.55	32.15	15.07

^a^Based on [12, 20, 53]. ^b^Based on this study. ^c^Considering the minimum and maximum dietary intake. ^d^Based on Jarosz [30]—(RDA for Cu, Fe, and Zn; AI for Mn)

Baked goods in Poland are consumed in larger amounts than other cereal products [27]. Based on the estimated share of baked goods in the dietary supply of microelements measured in the presented studies, baked goods cover the mineral requirement of an adult Pole: Cu in 49%, Mn in 52% (men)–66% (women), Fe in 17% (women)–30% (men), and Zn in 13% (men)–18% (women) (Table 6). Baked goods account for as much as about 90% of all Cu, Mn, Fe, and Zn consumed as cereal products in a daily diet (Table 5). Studies carried out in Poland in 2011 showed that a daily serving of bread supplies 77.8% RDA Cu, 25–45% RDA Fe, and 29–40% RDA Zn to an adult Pole, which also supports the statement that baked goods are a very important source of Cu in the diet [36]. Grembecka et al. [60] recounted that 100 g of bread supplied 9.57–20% RDA Fe and 5.41–15.3% RDA Zn to an adult Pole, depending on the type of bread (more in the case of whole meal bread). Studies carried out in Serbia revealed that bread and baked goods covered 10–22% of the daily requirement of Zn, 11–30% of Cu, and 17–37% of Fe [61]. UK studies showed that two slices of bread cover 14% of the daily requirement of Zn for women and 11% for men [58]. In Japanese diets, bread supplies ca. 3–4% Cu and Zn and 2.4–2.8% Mn [59].

**Table 6:** Share of respective groups of cereal products in the supply of Cu, Mn, Fe, and Zn in the diets of adult Poles

	Mean consumption (kg per month)^a^	Mean intake (mg per day)^b^			
		Cu	Mn	Fe	Zn
Breads + rolls	3.52	0.442	1.193	2.994	1.461
Groats + cereals	0.13	0.029	0.063	0.094	0.078
Pasta*	0.38	0.014	0.047	0.101	0.080
Rice*	0.16	0.006	0.021	0.026	0.038
Daily supply with cereal products (mg)^b^		0.492	1.324	3.215	1.657
		Share of respective groups of products in daily supply with cereal products (%) ^c^			
		Cu	Mn	Fe	Zn
Breads + rolls		89.98	90.11	93.11	88.18
Groats + cereals		5.914	4.764	2.918	4.686
Pasta*		2.858	3.522	3.152	4.852
Rice		1.252	1.581	0.816	2.294
		Contributions of some food groups to reference daily intakes (%)^d^			
Breads + rolls					
Women		49.09	66.28	16.63	18.26
Men		49.09	51.87	29.94	13.28
Groats + cereals					
Women		3.227	3.504	0.521	0.971
Men		3.227	2.742	0.938	0.706
Pasta*					
Women		1.559	2.591	0.563	1.005
Men		1.559	12.027	1.013	0.731
Rice*					
Women		0.683	1.163	0.146	0.475
Men		0.683	0.910	0.262	0.346

^a^Based on Statistical Yearbook of the Republic of Poland [27]; ^b^Based on this study; ^c^Daily supply with cereal products was adopted as 100%; ^d^Based on Jarosz [30]—(RDA for Cu, Fe and Zn; AI for Mn); *cooked pasta, groats, and rice (boiled without salt)

In the presented studies, the daily supply of microelements with breakfast cereals and groats amounts to 0.03 mg Cu, 0.06 mg Mn, 0.09 mg Fe, and 0.08 mg Zn, which accounts for 3.2% RDA, 2.7% AI (men)–3.5% AI (women), 0.5% RDA (women)–0.9% RDA (men), and 0.7% RDA (men)–0.7% RDA (women), respectively (Table 6). In the group of cereal products, the share of breakfast cereals and groats in supplying microelements accounts for 3% for Fe, approx. 5% for Zn and Mn, and 6% for Cu (Table 5). It should be taken into account that breakfast cereals are most often consumed with milk, which additionally increases their nutritional value and is particularly important for children who are the main consumers of breakfast cereals [29, 62]. Studies carried out in the UK showed that the diets of both adults and children consuming breakfast cereals are richer in Fe and Zn than the diets of people who do not eat such products [62]. In comparison to other cereal grains, buckwheat grains contain more Zn, Cu, and Mn of higher bioactivity [46]. Studies by Grembecka et al. [63] revealed that consumption of 100 g of buckwheat would supply approx. 17–21% RDA Zn and 10–12% RDA Fe, while 100 g of barley would account for 11–13% RDA Zn and 10–12% RDA Fe.

The average weekly consumption of pasta in Poland is less than 0.4 kg per person [27]. In a daily diet, such an amount of pasta supplies 1.6% RDA Cu, 2% AI Mn (men)–2.6% AI Mn (women), 0.6% RDA Fe (women)–1% RDA Fe (men), and 0.7% RDA Zn (men)–1% RDA Fe (women) (Table 6). Considering all cereal products, the share of pasta in the supply of Cu, Mn, Fe, and Zn is 2.8, 3.5, 3.2, and 4.9%, respectively (Table 6). Poles do not eat a lot of pasta, so the share of this product in the daily supply of microelements is not significant. However, in countries where pasta is consumed on a regular basis, it has a significant influence on the supply of minerals. Cubadda et al. [44] estimated that one serving of pasta available in the Italian market, weighing 80 g before boiling, would cover the daily mineral requirement (RDA) of an adult as follows: 18% Cu, 10–14% Zn (respectively for men and women), 5–9% Fe (respectively for women and men).

In Poland, on average, 0.16 kg rice is consumed per month (5.7 g per day). In a diet of an adult person, this product supplies 0.006 mg Cu, 0.021 mg Mn, 0.026 mg Fe, and 0.038 mg Zn, which accounts for 0.68% RDA, 0.91–1.16% AI (respectively for men and women), 0.26–0.15% RDA (respectively for women and men), and 0.35–0.48% RDA (respectively for men and women), respectively (Table 6). In comparison to other groups of cereal products, rice supplies approx. 1.3% Cu, 1.6% Mn, 0.8% Fe, and 2.3% Zn (Table 5). In the diets of Swedish people, the share of rice in covering the Recommended Dietary Intake (RDI) of microelements is 4.1% Cu, 0.6% Fe, 2.4% Mn, and 3.2% Zn (given the average intake of 11 g per day) [64]. In populations where rice is the basic food, the share of this cereal grain in covering the requirement of minerals is higher. In Vietnam, as much as 56% Mn and 49% Zn in the diet is sourced from rice [65]. According to Hashmi and Tianlin [66], one serving of rice (128 g) covers the mineral requirement of Malaysians as follows: 37–76% Fe (respectively for husked rice and brown rice), 30% Mn, 43% Cu, and 26% Zn. In the diets of the Japanese, white rice accounts for 23–30% Cu, 20–25% Zn, and 24–33% Mn, respectively, for women and men [59].

Despite the fact that cereal products contain large amounts of minerals, the assimilability of microelements deriving from such products is low, which is a result of the content of chelating agents such as fiber, phytic acid, oxalates, and polyphenols, while products of animal origin contain easily assimilable minerals. Hence, despite the supply of Cu being 27% higher in vegetarian diets, compared to consumers of products of animal origin, the level of Cu in the blood of vegetarians is lower [57]. Bioavailability of Cu from the diet amounts to ca. 50% [57]. It is also estimated that the bioavailability of Zn from the diet does not exceed 15% and is lower when this element is supplied from products of plant rather than of animal origin [67]. It is similar to Mn, of which less than 5% is absorbed from the diet [8]. Studies carried out in different countries did not show any effect of consuming cereal products, even those fortified with Fe, on the status of Fe in the body [68], which results from the fact that products of plant origin contain non-heme Fe which is only 1–8% assimilable, whereas heme Fe from products of animal origin is ca. 30% [69]. Studies by Krejpcio et al. [70] showed that when husk is removed from the caryopsis, bioavailability of Fe and Zn (but not Cu) in grains is increased. The authors explain that this is due to the fact that the husk contains most substances chelating minerals. In addition, bioavailability of microelements is affected by interactions between them. It was demonstrated that a deficiency of Fe inhibits the absorption of Cu, which is testified by simultaneous deficiency of these elements in people suffering from anemia [71]. In turn, excessive supply of Zn in a diet reduces the absorption of Cu [72]. What is more, it is suggested that the interaction can be mutual because a low level of Zn in blood serum is connected with a high level of Cu [72]. A low level of Fe in a diet increases the absorption of Mn, most likely in connection with the common mechanism of facilitated diffusion [73].

To sum up, cereal products are the main source of Cu, Mn, Fe, and Zn in the diets of Poles. Breakfast cereals contain particularly high levels of Mn, Fe, and Zn, whereas groats are rich in Cu. On the other hand, the highest share in supplying these microelements is that of baked goods with regard to the highest consumption of these products. This is connected with both the intake of such products and the content of microelements in them. In connection with low assimilability of minerals, they should not be considered the fundamental source of those microelements in the diets of Poles.
